# *Falsochrobactrum* *tianjinense* sp. *nov*., a New Petroleum-Degrading Bacteria Isolated from Oily Soils

**DOI:** 10.3390/ijerph191811833

**Published:** 2022-09-19

**Authors:** Mengjie Hu, Feifan Zhang, Gaoyuan Li, Haihua Ruan, Xinhao Li, Lei Zhong, Guanyi Chen, Yichao Rui

**Affiliations:** 1 School of Environmental Science and Engineering, Tianjin University, Tianjin 300350, China; 2 School of Biotechnology and Food Science, Tianjin University of Commerce, Tianjin 300134, China; 3 Rodale Institute, Kutztown, PA 19530, USA

**Keywords:** *Falsochbactrum*, hydrocarbon-degrading, oily soil, bacteria

## Abstract

The microbial remediation technology had great potential and attracted attention to total petroleum hydrocarbon pollution (TPH) remediation, but its efficiency is limited by its application in the field. In this study, a new TPH-degrading strain, TDYN1, was isolated from contaminated oil soil in Dagang Oilfield in Tianjin, China, and identified as *Falsochrobactrum* sp. by 16S rRNA sequence analysis. The physiological characterization of the isolate was observed. The orthogonal experiment was carried out for the optimum degradation conditions to improve its biodegradation efficiency. The strain was the gram-stain-negative, short rod-shaped, non-spore-forming, designated *Falsochrobactrum tianjinense* sp. *nov* (strain TDYN1); it had 3.51 Mb, and the DNA G + C content of the strain was 56.0%. The degradation rate of TDYN1 was 69.95% after 7 days of culture in optimal degradation conditions (temperature = 30 °C, pH = 8, salinity = 10 g L^−1^, petroleum concentration = 1 g L^−1^, and the inoculation dose of strain TDYN1 = 6%) and also reached more than 30% under other relatively extreme conditions. It suggested that the TDYN1 has great potential for TPH remediation in the soils of North China.

## 1. Introduction

Petroleum is an important energy source for human beings, and its consumption is increasing substantially with the development of society and urbanization. However, it is becoming one of the main contributors to soil pollution in petroleum extraction [1] and treatment processes. Due to the fact that petroleum hydrocarbons are macromolecular organic pollutants with complex components, they would eventually become difficult to degrade and pose a great threat to ecology and human health [2]. Therefore, there is an urgent need for environmentally efficient, friendly, and cost-effective remediation technologies.

Microbial bioremediation is a great application potential technique, in which microorganisms degrade hazardous organic pollutants into innocuous compounds, such as CO_2_, CH_4_, H_2_O, and biomass without adversely affecting the environment [3]. Because of its high efficiency, low cost, and environmental friendliness, bioremediation has become a promising approach for soil remediation. The success of microbial remediation depends on the effectiveness of the biodegradation of pollutants, for which there are many limiting factors in the process of microbial degradation, including the type of pollutants, the characteristics of the microorganisms themselves, and environmental conditions (such as pH, temperature, humidity, salinity, oxygen supply, and nutritional factors) [4].

It has been reported that different microbial species have different adaptation strategies to environmental factors [5,6]. For example, Liu et al. [7] (2016) reported that the degradation rate of bacterium Y-1 on crude petroleum was 44.8% at 35 °C, while the same was 60.2% at 55 °C. This indicated that the degradation rate of bacterial Y-1 was enhanced at a suitable temperature. Ma et al. [6] (2012) showed that *Pseudomonas* sp. JM2 was able to degrade more than 90% of fluorene and phenanthrene at 18 °C, 28 °C, and 37 °C, but less than 24% of fluorene and phenanthrene at 4 °C. It shows that microbial remediation technology can be effective only if it has its favorite environmental conditions or areas. Therefore, the identification of suitable environmental conditions can provide a basis for the application of microorganisms to remediate petroleum pollution.

Until now, more than 70 genera and 200 species of microorganisms have been found to degrade petroleum. The main petroleum hydrocarbon degraders are bacteria, fungi, and cyanobacteria, among which, the bacteria the most widely used include *Pseudomonas*, *Acinetobacter*, *Flavobacterium*, and *Corynebacterium* [8,9,10,11]. Ren et al. [12] isolated *Compostibacillus humi* from the soil in the north China oilfield area and found that when the initial concentration of petroleum was 0.5%, the strain could degrade 40.8% fluent within 20 days at 45 °C. Panda et al. [13] isolated *Pseudomonas aeruginosa* from the soil in the sewage irrigated area and found that when the initial concentration of petroleum was 0.5 g L^−1^, this could degrade by 49.93% fluent within 20 days. The isolated 60 bacterial strains from the soil in the sewage irrigated area and found that when the initial concentration of PAHs was 100 mg L^−1^, *Sphingomonas* 4C and *Sphingomonas* 23I could degrade by 87.2% fluent within 7 days at 30 °C, according to Zhou et al. [14]. However, for most of the studies, the degradation efficiency of microbiology is low and less than 60% [15,16]. Moreover, Northern China has a large number of oil fields and belongs to a temperate continental climate, the soil is neutral or alkaline, and the temperature is low in winter [17,18]. However, there are few studies on the screening of petroleum hydrocarbon-degrading bacteria in Northern China, and only some studies have reported that the degradation efficiency is low [7,17]. Thus, the application of microbial remediation of petroleum hydrocarbon pollution in Northern China is limited. Thus, and within the context of all the above, it is necessary to screen efficient petroleum degradation bacteria to be applied to petroleum-contaminated soil remediation in the Dagang oilfield.

Based on these reasons, the study collected the petroleum hydrocarbon-contaminated soil in the Dagang oil field, a typical oil-contaminated area in North China. The study aimed at screening and isolating the petroleum hydrocarbon-degrading bacteria from the oil-bearing soil. This would then be identified by 16S rRNA and analyzed for its physiological characteristics and ability on the petroleum hydrocarbon degradation. The optimal condition further promoted the bioremediation of petroleum hydrocarbon in North China.

## 2. Materials and Methodologies

### 2.1. Chemicals and Media

Oil-bearing soil was sampled from Dagang Oilfield (38° N, 117° E) in Tianjin, China in September 2018. Soil samples were collected by a five-point sampling method and refrigerated at 4 °C. Petroleum crude oil is heavy oil and collected from the Tianjin Dagang oil field.

The inorganic salt medium was as follows [19]:

- KH_2_PO_4_ 1.00 g L^−1^,
- K_2_HPO_4_ 1.00 g L^−1^,
- NaCl 10.00 g L^−1^,
- (NH_4_)_2_SO_4_ 1.50 g L^−1^,
- Anhydrous CaCl_2_ 0.10 g L^−1^,
- FeSO_4_·7H_2_O 0.01 g L^−1^; and,
- MgSO_4_ 0.20 g L^−1^.

For enrichment, 10 g of contaminated soil was added to 100 mL of inorganic salt medium containing 2% (*v*/*v*) petroleum [20]. The beef extract peptone medium was composed of 3 g L^−1^ of beef extract, 10 g L^−1^ of peptone, and 5 g L^−1^ of NaCl [21]. The inclined preservation medium (beef extract peptone solid medium) was composed of 5.00 g L^−1^ of beef extract, 10.00 g L^−1^ of peptone, 5.00 g L^−1^ of NaCl, and 20.00 g L^−1^ of agar. All media in this study were sterilized at 121 °C for 20 min before use. The pH of the diverse media utilized in this study was 7. 

### 2.2. Enrichment and Isolation of Oil-Bearing Soils Degrading Microorganisms

Ten grams of oily soil was placed in a conical flask containing 100 mL of beef extract peptone liquid culture medium, shaken on a constant temperature shaker at 30 ± 1 °C, 180 rpm, and enriched for 7 days. Two milliliters of enriched media was transferred into a fresh sample of the above medium and incubated under the same conditions described above. The strains were isolated from the above-enriched solution by a gradient dilution and spread plate technique. Then, different single colonies were selected and streaked in an LB plate. After multiple isolation and purification, a purified colony with a single form was obtained. The purified colonies were stored on a beef extract peptone solid medium at 4 °C.

### 2.3. Identification and Genome Features of Strains

The bacterial genomic DNA was extracted using the AxyPrep DNA isolation kit.

The universal bacterial 16S rRNA primers, 27F (5′-AGAGTTTGATCCTGGCTCAG-3′) and 1492R (5′-CTACGGCTACCTTGTTACGA-3′) were used to amplify bacterial 16S rDNA [22]. The PCR product of purified strains was subjected to DNA sequencing by the sequencerABI3730-XL. The NCBI Blast program was used to compare the spliced sequence files with the data in the NCBI 16S rRNA database (https://www.ncbi.nlm.nih.gov (accessed on 5 September 2021)). The species information with the greatest similarity to the sequences to be tested was obtained, which was the identification result [7]. Phylogenetic trees for 16S rRNA were built using MEGA 6.0 software (Arizona State University, Tempe, AZ, USA).

The whole-genome sequence of strain TDYN1T was determined by using an Illumina Hiseq 4000 platform by Majorbio (genome sequencing depth, 100×; paired-end read length, 150 bp). De novo assembly of raw reads was performed with SOAPdenovo Version 2.04 (http://soap.genomics.org.cn/ (accessed on 12 November 2021)). The DNA G + C content was calculated using the Bowtie2 Version 2.29 (http://bowtie-bio.sourceforce.net/bowtie2/index.shtml (accessed on 12 November 2021 )).

### 2.4. Physiological Characterization of the Isolated Strain

The TDYN1 was inoculated on beef paste peptone solid plate medium and incubated under a constant temperature incubator at optimal growing conditions for 2 days in an inverted position. Some of its physiological characteristics were analyzed using the following working approaches:

- Electron microscopy [23],
- Gram staining observation [24],
- Oxidase activity observation,
- Peroxidase activity observation [25],
- Sugar fermentation observation,
- Ammonia production observation; and,
- Indole observation.

To verify some of the physiological characteristics of the strains.

### 2.5. TPH Biodegradation Ability of Falsochrobactrum sp. TDYN1

To determine the degradation ability of the isolate to petroleum, the isolate was cultured at 30 °C and 150 rpm for 24 h (OD_600_: 1) in the beef extract peptone medium. Then, the bacterial solution was placed in a 50-mL centrifuge tube at 6000 rpm for 10 min. The centrifuged cells were washed with normal saline and centrifuged 2 to 3 times to be reserved. The experimental group was inoculated with 3 mL of bacterial solution (normal saline strain OD_600_: 1) in 100 mL of inorganic salt medium containing 0.1 g of crude petroleum and shaken at 30 °C and 180 rpm for 7 days at a constant temperature, which was employed for biodegradation tests. The remaining crude petroleum was extracted several times with a 50-mL petroleum riddle in small amounts until it was completely extracted and transferred to a 100-mL volumetric flask to fix the volume to 100 mL. Using a UV spectrophotometer, the absorbance value of the strain was measured at the maximum absorption wavelength of petroleum, and the degradation efficiency of the strain was judged against the standard curve. The experimental procedure was set up with blank control and three parallel tests at the same time. The degradation rate (expressed as %) of crude petroleum was calculated by the following formula:

$$TPHs\;removal\;\left(\%\right)=\frac{M0-M}{M0}\times 100\%$$

where:

- *M*0 is the weight of petroleum initially added into the medium; and,
- *M* is the weight of residual petroleum after extraction.

Through orthogonal experimental design, the degradation conditions of the degrading bacteria are optimized to improve their degradation efficiency. The main experimental design and stepwise procedure are as follows:

The petroleum-inorganic salt liquid medium was used as the basic medium, and four levels were selected for each of the five factors that need to be considered: viz. temperature, pH, salinity, petroleum concentration, and the inoculum dose of strain TDYN1. According to the orthogonal test L16 (45), five factors and four levels [26] need to be used. The degradation conditions of the degrading bacteria were optimized. The selected levels of each factor are shown in Table 1, and the optimized experimental design scheme is shown in Table 2. Each group was cultured for 7 d, and each group was repeated 3 times, and the obtained oil degradation rate was averaged.

## 3. Result and Discussion

### 3.1. Isolation and Identification of the Falsochrobactrum TDYN1

**Figure 1:**
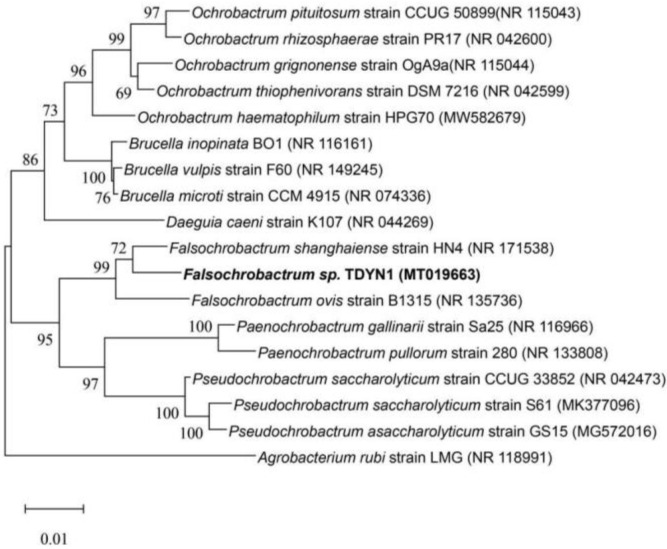
Phylogenetic trees of *Falsochrobactrum* sp. TDYN1. Phylogenetic trees were constructed based on the 16S rRNA gene sequences (1371 bp) using the neighbor-joining method. The phylogeny test used the bootstrap method with 1000 replications.

**Figure 2:**
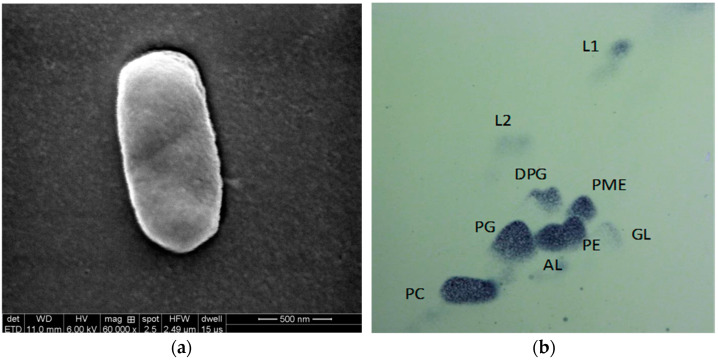
(**a**) Scanning electron microscopy (SEM) of strain TDYN1; (**b**) polar lipid profile of strain TDYN1.

The TDYN1 strain was isolated from the petroleum-bearing soils, which was selected from multiple isolates after multiple isolation and purification. The isolate was identified by gene sequencing, and the complete 16S rRNA sequence of TDYN1 was determined to be 1371 bp (PCR picture of strain TDYN1 could be found in Figure S1). The sequence of strain TDYN1 was searched in NCBI Blast, and the results indicated that the strain TDYN1 belongs to the genus *Falsochrobactrum* sp. The strain TDYN1 had the highest sequence similarity to *Falsochrobactrum shanghainese* HN4^T^ [27] (98.3%) and *Falsochrobactrum Ovis* B1315^T^ [28] (97.2%), followed by *Ochrobactrum* (96.2%); other species shared below 90% sequence similarity. The results showed that the strains TDYN1, strains HN4^T^ [27], and B1315^T^ [28] were all homologous strains, but belonged to different branches (Figure 1). This topology was determined by the maximum likelihood tree and the minimum evolution tree at the same time (Figures S3 and S4) and indicated that the strain TDYN1T may belong to the genus *Falsochrobactrum* sp. and represent a new species entirely. The gene accession number of the strain TDYN1 is MT019663 on the NCBI official website. Although the *Falsochrobactrum* was first found in the year of 2013, there were only two strains that have been reported (*Falsochrobactrum ovis* sp. *nov*, *Falsochrobactrum shanghainese* sp. *nov.*) [27,28]. However, all these studies did not report that these strains could degrade TPH. Our results suggest that the strain TDYN1 has the potential to degrade petroleum by the analysis of the phylogenetic tree. It was not only because the strain was screened and isolated from petroleum-contaminated soil and petroleum-inorganic salt liquid medium, respectively, but the result also showed that the *Falsochrobactrum* is closely related to *Ochrobactrum* by the analysis of the phylogenetic tree (Figure 2). The *Ochrobactrum* has been widely reported to be adept at degrading TPH [29,30], e.g., *Ochrobactrum cytisi*, *Ochrobactrum halosaudia* AJH1, *Ochrobactrum ciceri* and so on [31,32,33]. Therefore, we speculate that the genus *Falsochrobactrum* TDYN1 has the potential to degrade petroleum.

### 3.2. Physiological Characterization of the Isolated Strain

After scanning by electron microscopy, the bacterium showed a short rod shape without flagella (Figure 2a). The cells of the TDYN1 were red after being stained by the gram, which showed that TDYN1 was a gram-negative bacterium. Methyl red and methyl acetomethanol results were negative. Glucose and lactose tests were acid-producing but not gas-producing. The indole test was positive. The test of catalase was positive by the foaming method. The 1% aqueous solution of dimethyl p-phenylenediamine hydrochloride and the 1% a-phenolphthalein alcohol (95%) solution (mixed) were positive for the oxidase test. The G + C content of chromosomal DNA was determined by sequencing sequence data. The chromosomal DNA G + C content of the newly isolated strain TDYN1 was 56.0%. TDYN1 was the same in morphology and taxonomic shape as its strains of the same genus and had similarities with HN4^T^ strains, except for the difference in mobility (Table 3).

Strain TDYN1 differs from closely related *Falsochrobactrum ovis* B1315^T^ and *Falsochrobactrum* *shanghainese* HN4^T^ grown under the same conditions in the composition of principal fatty acids. The main fatty acids of strain TDYN1 were summarized as C18.1N9C (43%) and C16.0 (28%), followed by C18.0 (19%), C16.1 (5%), and C17.0 (3%). In comprise, the main fatty acids of *Falsochrobactrum ovis* B1315^T^ and *Falsochrobactrum shanghainese* HN4^T^ have a huge difference. Fatty acids are shown in Table 4.

The polar lipid profile consisted of the major compounds diphosphatidylglycerol (DPG), phosphatidylmonomethylethanolamine (PME), phosphatidylethanolamine (PE), phosphatidylglycerol (PG), phosphatidylcholine (PE) and the unidentified amino lipid (AL), the unidentified glycolipid (GL), and unidentified polar lipids (L1-2) (Figure 2b). The quinone system was ubiquinone Q-10 (74.6%), Q-9 (19.0%), Q-8 (6.4%). The detailed polar lipid data are listed in Table 5.

The results of phylogenetic, phenotypic, and chemotaxonomic traits classifying showed that the TDYN1 strain belonged to *Falsochrobactrum* sp. and was different from related species of the genus *Falsochrobactrum* sp. The genome size was 3,510,529 bp with K-mer coverage 352.5× and 29 contigs. Annotation of the contigs identified 3465 coding sequences (CDSs), 3 rRNAs, and 46 tRNAs. The DNA G + C content of the strain TDYN1 was 56.0%, which was lower than that of *Falsochrobactrum shanghaiense.* HN4^T^ (56.9%) but higher than that of Falsochrobactrum ovis. B1315^T^ (49.1%). This Whole Genome Shotgun project had been deposited at DDBJ/ENA/GenBank under the accession JAHRVA000000000. The version described in this paper is with regard to the JAHRVA010000000. The strain TDYN1 showed without flagella, but the strain HN4^T^ showed with flagella [27]. The strain TDYN1 is different from strains HN4^T^ and B1315^T^ in genomic information and physiological characteristics. Therefore, the strain TDYN1 was represented as a new species and proposed *Falsochrobactrum tianjinnese* sp. *nov.* (Strain TDYN1). The new petroleum-degrading bacteria was preserved in the China General Microbiological Culture Collection Center, China Center for Typical Cultures Preservation, and Korean Collection for Type Cultures (Figures S4–S6).

### 3.3. TPH Biodegradation Ability and Optimal Degradation Conditions of Strain TDYN1

**Figure 3:**
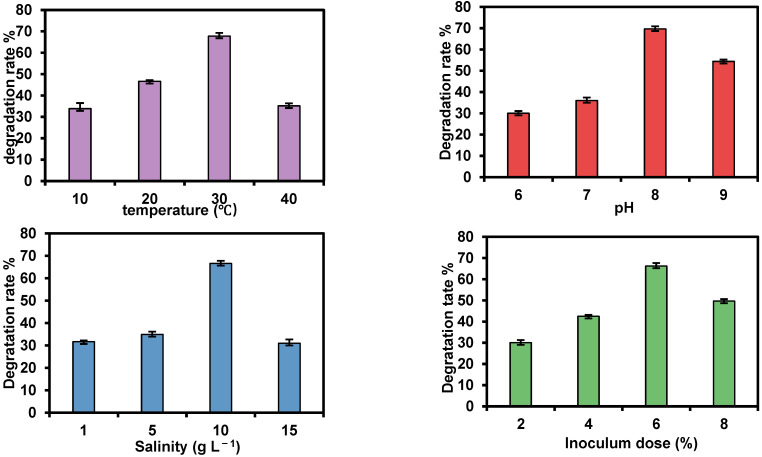

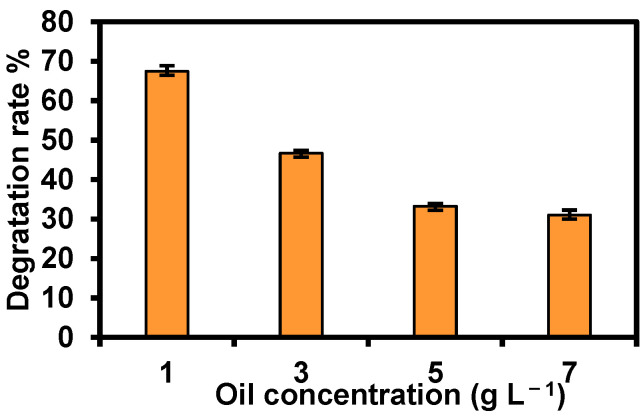
Effects of different culture conditions on the degradation rate of strain.

The optimal degradation conditions of strain TDYN1 were analyzed by the orthogonal test with 5 factors and 4 levels (Table 6). The results showed that the 10th group (A3B2C1D3E1) of the orthogonal test had the highest degradation rate at a temperature of 30 °C, a pH of 7.0, and salinity of 1 g L^−1^. The inoculum dose of stain TDYN1 was 6%, and the petroleum concentration was 1 g L^−1^; in this condition, the degradation rate of the strain reached a level of 67.95%. Compared with the range analysis, it can be found that the R-value of petroleum concentration among the five factors is 16.18, which has the greatest influence on the effect of TPH degradation. The order of the primary and secondary factors affecting the 5 factors is petroleum concentration > pH > inoculum dose > salinity > temperature. Further analysis of variance can find that the displayed results are consistent with the above (Table 7). The optimal theoretical conditions for petroleum degradation after optimization are as follows:

- temperature = 30 °C,
- initial pH = 8,
- salinity = 10 g L^−1^,
- petroleum concentration = 1 g L^−1^; and,
- the inoculation dose of strain = 6% (Table 6 and Figure 3).

In this condition, the petroleum degradation rate reached the highest point at 69.95% in 7 days by TDYN1. It is higher than many strains reported before, such as by Jiang [34] et al., who isolated 6 strains in the polluted seawater of the South China Sea and found that the petroleum degradation rate is 20–55%; Liu [15] et al. isolated three strains of degrading bacteria in petroleum-contaminated soil, and the petroleum degradation rate of single strain was lower than 45% after 7 days. Therefore, our results show that TDYN1 had a great commercial and application potential for application on petroleum hydrocarbon pollution remediation.

On the other hand, the TDYN1 showed excellent degradation ability under relatively extreme conditions. In our results, the TDYN1 could still degrade TPH under the conditions of low temperature, high temperature, high salt, and high pollutant concentration (Figure 3). For example, when the temperature exceeded 30 °C, the degradation rate decreased but still reached 33.85% at 10 °C. At the same time, the solution rate can still reach more than 31% at 15 g L^−1^ high salinity. A high degradation rate also can be achieved under the condition of 7 g L^−1^ crude petroleum. It indicates that the strain can tolerate high salt and high concentration of crude petroleum. Normally, most petroleum hydrocarbon-degrading strains can only maintain degradability under pH 7; both acidic and alkaline conditions will inhibit the growth and activity of microorganisms; high salinity will also cause a sharp decrease in the number and viability of microorganisms [35]. The degradability of the strains would be significantly decreased or increased by environmental changes [4]. Recently, many studies have also reported that the TPH degradation bacteria could adapt to relatively extreme environments, e.g., high salinity [36], low temperature [6], and so on. However, these strains are still not enough to be applicable in soil remediations due to the various soil environment and climatic conditions. Therefore, our study showed that the TDYN1 had strong adaptability to the environment, especially in relative extreme temperatures and salinized soil conditions. Its good degradation efficiency indicated the strain could have great potential for TPH remediation in the soils of North China.

Although the strain was proven to have a high potential for TPH remediation in the soils, the industrial application of the strain also has many limitations. For example, the strain might have difficulty surviving or exhibit low activity in contaminated soil due to soil pollution or poor environmental surroundings [37,38]. Therefore, it is crucial to improve the adaptability of this strain to the complex soil environment; as such, further experiments are required to clarify its remediation abilities and deficiencies. This provides the important theoretical support necessary for the application of the strain. On the other hand, a large number of studies have shown that the efficiency and application prospect of microbial remediation technology can be improved by some compound means, such as the addition of nutrient solution [39], the fixation of biochar as a carrier [40,41], and the development of highly efficient engineered bacteria [42]. It is possible to enhance the prospects of industrial application of this strain and microbial remediation technology in the field of soil remediation. 

## 4. Conclusions

In conclusion, the new petroleum degradation strain *Falsochrobactrum tianjinnese* sp. *nov.* had been isolated and found. The gram-stain-negative, short rod-shaped, non-spore-forming was designated *Falsochrobactrum* sp. TDYN1. The N50 and N90 values of TDYN1 were 462,105 bp and 229,496 bp, respectively. Its Whole Genome Shotgun project had been deposited at DDBJ/ENA/GenBank under the accession JAHRVA000000000. The TPH degradation rate of TDYN1 was 69.95% after 7 days at the optimal degradation conditions (temperature = 30 °C, pH = 8, salinity = 10 g L^−1^, petroleum concentration = 1 g L^−1^, and the inoculation dose of strain TDYN1 = 6%) and also reached more than 30% under other relatively extreme conditions. It suggested that TDYN1 had strong adaptability to the environment, especially in relatively low temperatures or salinized soil conditions, which indicated the strain could have great potential for TPH remediation in the soils of North China. However, there is still a lot of work to be done for its industrialized application, e.g., analyzing the main degraded petroleum hydrocarbon components of the strain TDYN1, carrying out the degradation experiment of the strain TDYN1 in petroleum hydrocarbon-contaminated soil, and so on.

## Figures and Tables

**Table 1:** Design of environmental factors and level of orthogonal test.

Levels	Factor				
	(A) Temperature	(B) pH	(C) Salinity	(D) Inoculum Dose	(E) Petroleum Concentration
1	10 °C	6	1 g L^−1^	2%	1 g L^−1^
2	20 °C	7	5 g L^−1^	4%	3 g L^−1^
3	30 °C	8	10 g L^−1^	6%	5 g L^−1^
4	40 °C	9	15 g L^−1^	8%	7 g L^−1^

**Table 2:** Optimization scheme of orthogonal test.

Number	A	B	C	D	E
1	1	1	1	1	1
2	1	2	2	2	2
3	1	3	3	3	3
4	1	4	4	4	4
5	2	1	2	3	4
6	2	2	1	4	3
7	2	3	4	2	2
8	2	4	3	1	1
9	3	1	3	4	2
10	3	2	1	3	1
11	3	3	4	2	4
12	3	4	2	1	3
13	4	1	4	2	3
14	4	2	3	1	4
15	4	3	2	4	1
16	4	4	1	3	2

**Table 3:** Physiological characteristics of strain TDYN1 and the species of the genus *Falsochrobactrum*. Strains: 1, TDYN1; 2, *Falsochrobactrum ovis* B1315^T^ [28]; 3, *Falsochrobactrum shanghaiense* HN4^T^ [27]; +, positive; −, negative, ND, no data. The results of TDYN1 were from this study, other data were from the references.

Characteristic		1	2	3
Gram stain		−	−	−
Shape		rod	rod	Short−rod
Motile		−	−	+
Spore		+	−	−
Capsule		+	ND	ND
Glucose	Acid production	+	ND	ND
	Aerogenesis	−	ND	ND
Lactose	Acid production	+	ND	ND
	Aerogenesis	−	ND	ND
Methyl red		−	ND	ND
Methyl acetyl alcohol		−	ND	ND
Benzpyrole		+	ND	ND
Oxidase		+	+	+
Catalase		+	ND	ND
Production of ammonia		+	ND	ND
DNA G + C content (mol%)		56.0%	49.1%	56.9%
pH		7.0–7.4	ND	7.0
Temperature		30 ± 1 °C	30 °C	30–35 °C
NaCl		1%	ND	1%

**Table 4:** Cellular fatty acid profiles of strain TDYN1 and its relatives. Strains: 1, TDYN1; 2, *Falsochrobactrum ovis* B1315^T^ [28]; 3, *Falsochrobactrum shanghainese* HN4^T^ [27]; ND, no data. The results of TDYN1 were from this study, other data were from the references.

Fatty Acid	1	2	3
C_8.0_	3.6	ND	ND
C_10.0_	3.6	ND	ND
C_12.0_	20.1	ND	ND
C_13.0_	5.6	ND	ND
C_14.0_	318.2	ND	ND
C_15.0_	179.3	ND	ND
C_15.1_	8.5	12	ND
C_16.0_	10,733.9	ND	8.6
C_16.1_	1896.5	ND	ND
C_17.0_	1195.3	ND	ND
C_17.1_	99.7	9.6	ND
C_18.0_	7282.6	ND	13.5
C_18.1_N9C	16,543.4	ND	ND
C_18.1_N9T	4.5	ND	ND
C _18:1_ 2-OH	ND	ND	3.2
C_18.2_N6C	48.1	ND	ND
C_18:1_ω7c	ND	32.1	ND
C_19:0_cycloω8c	ND	44.4	30.5
C_20.0_	14.6	ND	ND
C_20.1_	65.4	ND	ND
C_20:2_ω6.9c	ND	1.9	ND
C_20.4_N6	4.1	ND	ND
C_22.0_	7.5	ND	ND
C_22.1_N9	349.2	ND	ND
C_22.2_	8.9	ND	ND

**Table 5:** Cellular polar lipids profiles of strain TDYN1 and its relatives. Strains: 1, TDYN1; 2, *Falsochrobactrum ovis* B1315^T^ [28]; 3, *Falsochrobactrum shanghainese* HN4^T^ [27]; +, positive; ND, no data. The results of TDYN1 were from this study, other data were from the references.

Polar Lipids	1	2	3
Diphosphatidylglycerol	+	+	+
Phosphatidylglycerol	+	+	+
Phosphatidylethanolamine	+	+	+
Phosphatidylmonomthylethanolamine	+	+	ND
Phosphatidylcholine	+	+	+
Unidentified aminolipid AL	+	ND	ND
Unidentified aminolipid AL1	ND	+	ND
Unidentified glycolipid GL	+	ND	ND
Unidentified glycolipid APL1, 2	ND	ND	+
Unidentified phospholipid PL7	ND	+	ND
Unidentified phospholipid GL1, 2	ND	ND	+
Unidentified polar lipids L1-2	+	ND	ND
Unidentified lipid	ND	ND	+
Unidentified aminophospholipid L1, 2, 3	ND	ND	+

**Table 6:** Orthogonal test results for direct analysis. A is temperature, B is pH, C is salinity, D is the inoculation dose of strain, E is petroleum hydrocarbon concentration, Ki is the sum of the decreasing solution rates of all factors I, and R is range.

Number	A	B	C	D	E	Average Degradation Rate (%)
1	1	1	1	1	1	6.72 ± 0.24
2	1	2	2	2	2	29.36 ± 1.21
3	1	3	3	3	3	59.34 ± 0.51
4	1	4	4	4	4	39.96 ± 1.54
5	2	1	2	3	4	28.67 ± 0.23
6	2	2	1	4	3	23.29 ± 1.18
7	2	3	4	2	2	42.84 ± 0.56
8	2	4	3	1	1	61.71 ± 0.93
9	3	1	3	4	2	43.01 ± 1.66
10	3	2	1	3	1	67.95 ± 0.71
11	3	3	4	2	4	33.03 ± 1.40
12	3	4	2	1	3	29.38 ± 0.51
13	4	1	4	2	3	34.73 ± 0.82
14	4	2	3	1	4	22.35 ± 0.44
15	4	3	2	4	1	52.34 ± 0.50
16	4	4	1	3	2	27.72 ± 1.00
K1	135.38	113.13	125.68	120.16	188.72	
K2	146.51	142.95	139.75	129.96	132.93	
K3	173.37	177.55	186.41	183.68	146.74	
K4	137.14	158.77	140.56	158.6	124.01	
R	9.06	16.11	11.46	15.88	16.18	
optimal conditions	A3	B3	C3	D3	E1	

**Table 7:** Variance analysis of orthogonal test where A–E represent temperature, pH, salinity, inoculation dose of strain, and petroleum hydrocarbon concentration, respectively, and such parameters as degree of freedom, the mean square, and where F: F-value; **: *p* < 0.01 (F > F0.01 (3.51)) and *: *p* < 0.05 (F > F0.05 (3.51)) are clearly stated.

Source	Sum of Square	df	Mean Square	F	*p*	Significant
A	871.239	3	290.413	160.423	0.000	**
B	1679.998	3	559.999	309.341	0.000	**
C	5259.636	3	1753.212	968.466	0.000	**
D	4868.000	3	1622.667	896.353	0.000	**
E	5410.477	3	1803.492	996.241	0.000	**
error	57.930	32	1.810			
total	79,793.420	48				

## Supplementary Materials

The following supporting information can be downloaded at: https://www.mdpi.com/article/10.3390/ijerph191811833/s1, Figure S1. PCR picture of strain TDYN1; Figure S2. The evolutionary history was inferred by using the Maximum Likelihood method based on the Tamura-Nei model. The tree with the highest log likelihood (−28168.99) is shown. Initial tree(s) for the heuristic search were obtained automatically by applying Neighbor-Join and BioNJ algorithms to a matrix of pairwise distances estimated using the Maximum Composite Likelihood (MCL) approach, and then selecting the topology with superior log likelihood value. The tree is drawn to scale, with branch lengths measured in the number of substitutions per site. The analysis involved 18 nucleotide sequences. All positions containing gaps and missing data were eliminated. There were a total of 1310 positions in the final dataset. Evolutionary analyses were conducted in MEGA7; Figure S3. The evolutionary history was inferred using the Minimum Evolution method. The optimal tree with the sum of branch length = 29.94657966 is shown. The tree is drawn to scale, with branch lengths in the same units as those of the evolutionary distances used to infer the phylogenetic tree. The evolutionary distances were computed using the Maximum Composite Likelihood method and are in the units of the number of base substitutions per site. The ME tree was searched using the Close-Neighbor-Interchange (CNI) algorithm at a search level of 1. The Neighbor-joining algorithm was used to generate the initial tree. The analysis involved 18 nucleotide sequences. All positions containing gaps and missing data were eliminated. There were a total of 1310 positions in the final dataset. Evolutionary analyses were conducted in MEGA7; Figure S4. Culture collection certificates from CCTCC; Figure S5. Culture collection certificates from CGMCC; Figure S6. Culture collection certificates from KCTC.
