# Supplementary material for: Falsochrobactrum tianjinense sp. nov., a New Petroleum-Degrading Bacteria Isolated from Oily Soils
Source: Int J Environ Res Public Health. 2022 Sep 19;19(18):11833. doi: 10.3390/ijerph191811833 (PMC9517009; doi:10.3390/ijerph191811833)
Supplement: Supplementary file 1 [file ijerph-19-11833-s001.zip › ijerph-1879063-supplementary.pdf]

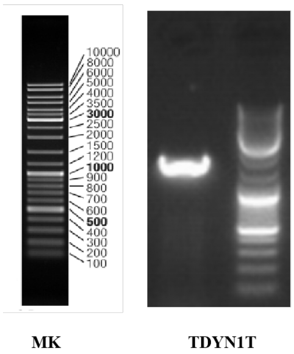

Figure S1. PCR picture of strain TDYN1

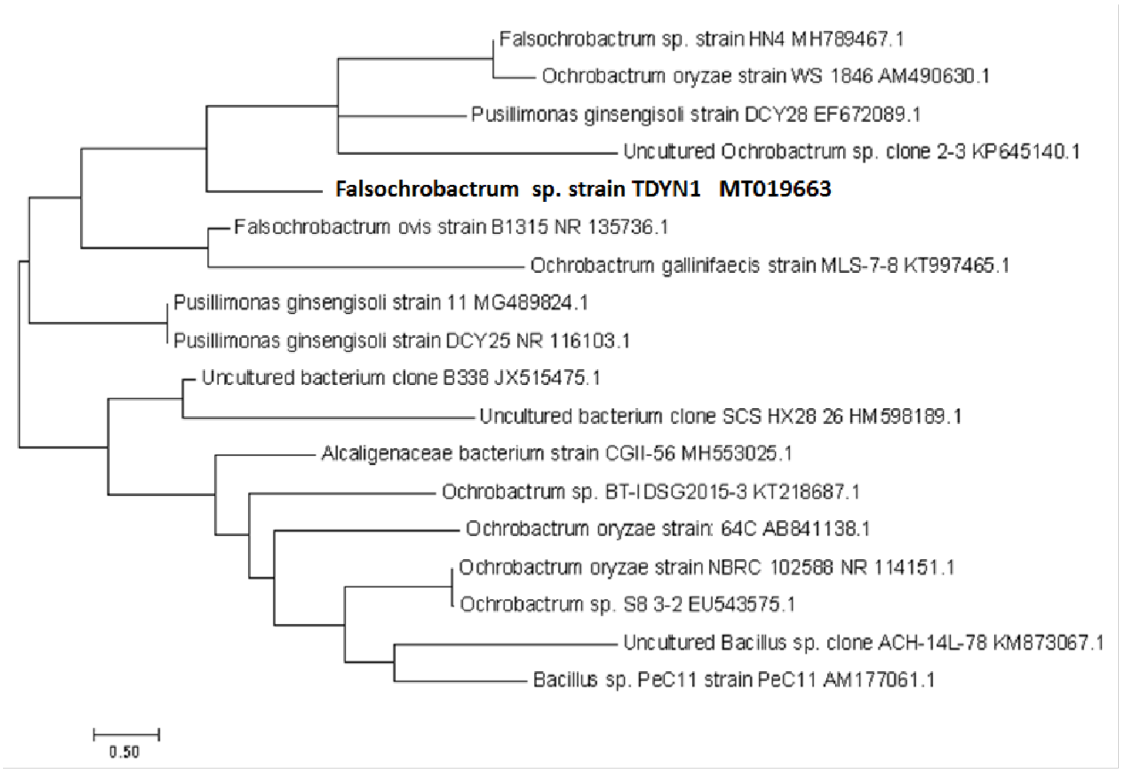

Figure S2. The evolutionary history was inferred by using the Maximum Likelihood method based on the Tamura-Nei model. The tree with the highest log likelihood (-28168.99) is shown. Initial tree(s) for the heuristic search were obtained automatically by applying Neighbor-Join and BioNJ algorithms to a matrix of pairwise distances estimated using the Maximum Composite Likelihood (MCL) approach, and then selecting the topology with superior log likelihood value. The tree is drawn to scale, with branch lengths measured in the number of substitutions per site. The analysis involved 18 nucleotide sequences. All positions containing gaps and missing data were eliminated. There were a total of 1310 positions in the final dataset. Evolutionary analyses were conducted in MEGA7.

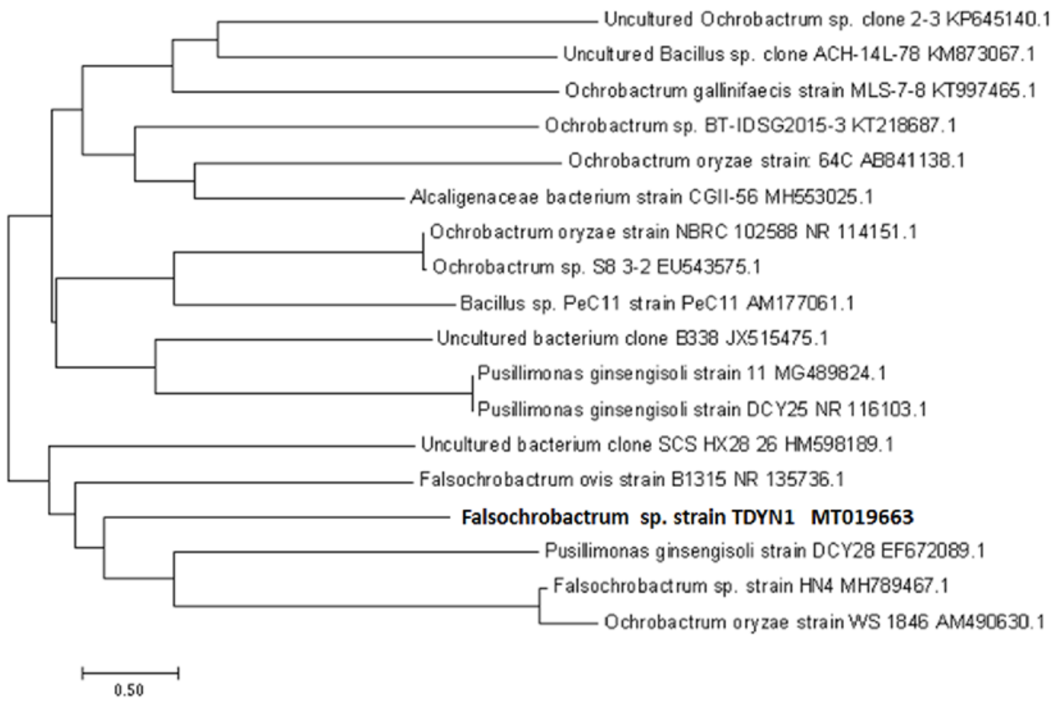

Figure S3. The evolutionary history was inferred using the Minimum Evolution method. The optimal tree with the sum of branch length = 29.94657966 is shown. The tree is drawn to scale, with branch lengths in the same units as those of the evolutionary distances used to infer the phylogenetic tree. The evolutionary distances were computed using the Maximum Composite Likelihood method and are in the units of the number of base substitutions per site. The ME tree was searched using the Close-Neighbor-Interchange (CNI) algorithm at a search level of 1. The Neighbor-joining algorithm was used to generate the initial tree. The analysis involved 18 nucleotide sequences. All positions containing gaps and missing data were eliminated. There were a total of 1310 positions in the final dataset. Evolutionary analyses were conducted in MEGA7.

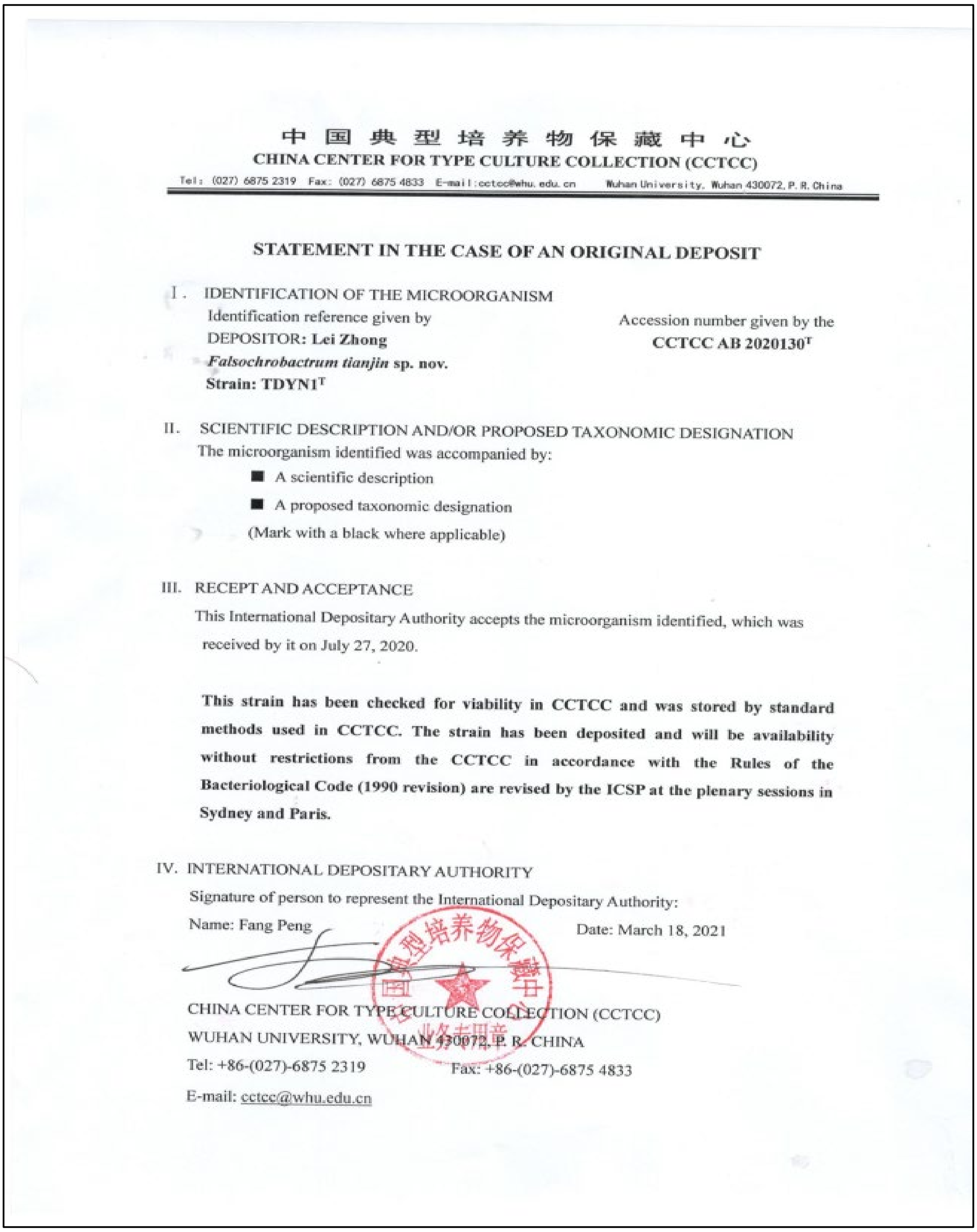

中国典型培养物保藏中心

CHINA CENTER FOR TYPE CULTURE COLLECTION (CCTCC)

Tel: (027) 6875 2319 Fax: (027) 6875 4833 E-mail:cctcc@whu.edu.cn Wuhan University, Wuhan 430072, P. R. China

STATEMENT IN THE CASE OF AN ORIGINAL DEPOSIT

I. IDENTIFICATION OF THE MICROORGANISM

Identification reference given by
DEPOSITOR: **Lei Zhong**
Falsochrobactrum tianjin **sp. nov.**
Strain: TDYN1^T

Accession number given by the
CCTCC AB 2020130^T

II. SCIENTIFIC DESCRIPTION AND/OR PROPOSED TAXONOMIC DESIGNATION

The microorganism identified was accompanied by:

- ■ A scientific description
- ■ A proposed taxonomic designation

(Mark with a black where applicable)

III. RECEPT AND ACCEPTANCE

This International Depositary Authority accepts the microorganism identified, which was received by it on July 27, 2020.

This strain has been checked for viability in CCTCC and was stored by standard methods used in CCTCC. The strain has been deposited and will be availability without restrictions from the CCTCC in accordance with the Rules of the Bacteriological Code (1990 revision) are revised by the ICSP at the plenary sessions in Sydney and Paris.

IV. INTERNATIONAL DEPOSITARY AUTHORITY

Signature of person to represent the International Depositary Authority:

Name: Fang Peng

Date: March 18, 2021

CHINA CENTER FOR TYPE CULTURE COLLECTION (CCTCC)
WUHAN UNIVERSITY, WUHAN 430072, P. R. CHINA
Tel: +86-(027)-6875 2319 Fax: +86-(027)-6875 4833
E-mail: cctcc@whu.edu.cn

Figure S4. Culture collection certificates from CCTCC

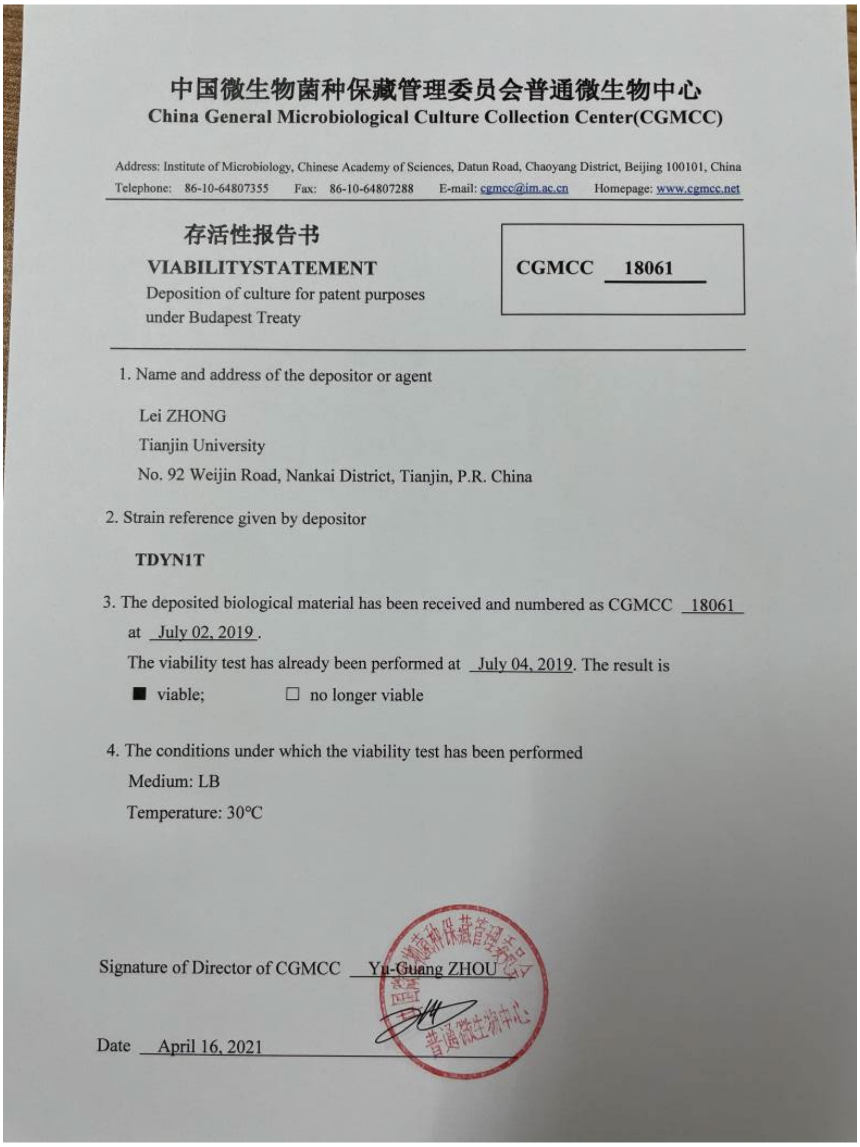

中国微生物菌种保藏管理委员会普通微生物中心
China General Microbiological Culture Collection Center(CGMCC)

Address: Institute of Microbiology, Chinese Academy of Sciences, Datun Road, Chaoyang District, Beijing 100101, China
Telephone: 86-10-64807355 Fax: 86-10-64807288 E-mail: cgmcc@im.ac.cn Homepage: www.cgmcc.net

存活性报告书
VIABILITYSTATEMENT
Deposition of culture for patent purposes
under Budapest Treaty

CGMCC 18061

1. Name and address of the depositor or agent

Lei ZHONG
Tianjin University
No. 92 Weijin Road, Nankai District, Tianjin, P.R. China

2. Strain reference given by depositor

TDYN1T

3. The deposited biological material has been received and numbered as CGMCC 18061 at July 02, 2019.
The viability test has already been performed at July 04, 2019. The result is
■ viable; ☐ no longer viable

4. The conditions under which the viability test has been performed
Medium: LB
Temperature: 30℃

Signature of Director of CGMCC Yu-Guang ZHOU

Date April 16, 2021

Figure S5. Culture collection certificates from CGMCC.

Korean Collection for Type Cultures (KCTC)
Korea Research Institute of Bioscience and Biotechnology (KRIBB)
181 Ipsin-gil, Jeongeup-si, Jeonbuk 56212, South Korea
Tel: +82-63-570-5602, FAX: +82-63-570-5609
E-mail: deposit@kribb.re.kr

Certificate of Deposit

Ref.: 20210689 Date of issue: JUN 11, 2021

Taxonomic designation	:	*Falsochrobactrum* strain
Accession number	:	KCTC 82789
Depositor(s)	:	Lei Zhong
Strain code by the depositor(s)	:	TDYN1

The above microorganism has been successfully deposited into the general collection of microorganism of the Korean Collection for Type Cultures (KCTC) and confirmed the identity of the microorganism under this KCTC number.
This microorganism will be available without restrictions for research and academic purposes in the publicly accessible section of the KCTC. It will be included in published and online catalogues after publication of this number by the authors.

Song-Gun Kim

Curator of Bacteria
Song-Gun Kim Ph.D.

Telephone: +82-63-570-5600
Fax: +82-63-570-5609
E-mail: sgkim@kribb.re.kr
web: https://kctc.kribb.re.kr

Figure S6. Culture collection certificates from KCTC
